# Identification of Male Gametogenesis Expressed Genes from the Scallop *Nodipecten subnodosus* by Suppressive Subtraction Hybridization and Pyrosequencing

**DOI:** 10.1371/journal.pone.0073176

**Published:** 2013-09-16

**Authors:** Raúl Llera-Herrera, Alejandra García-Gasca, Cei Abreu-Goodger, Arnaud Huvet, Ana M. Ibarra

**Affiliations:** 1 Aquaculture Genetics and Breeding Laboratory, Centro de Investigaciones Biológicas del Noroeste, La Paz, Baja California Sur, Mexico; 2 Centro de Investigación en Alimentación y Desarrollo, Mazatlán, Sinaloa, Mexico; 3 Laboratorio Nacional de Genómica para la Biodiversidad (Langebio), Centro de Investigación y Estudios Avanzados del Instituto Politécnico Nacional (CINVESTAV-IPN), Irapuato, Guanajuato, Mexico; 4 Laboratoire des Sciences de l'Environnement Marin, Institut Français de Recherche pour l'Exploitation de la Mer, (IFREMER), Centre de Bretagne, Plouzané, France; Universitat de Barcelona, Spain

## Abstract

Despite the great advances in sequencing technologies, genomic and transcriptomic information for marine non-model species with ecological, evolutionary, and economical interest is still scarce. In this work we aimed to identify genes expressed during spermatogenesis in the functional hermaphrodite scallop *Nodipecten subnodosus* (Mollusca: Bivalvia: Pectinidae), with the purpose of obtaining a panel of genes that would allow for the study of differentially transcribed genes between diploid and triploid scallops in the context of meiotic arrest and reproductive sterility. Because our aim was to isolate genes involved in meiosis and other testis maturation-related processes, we generated suppressive subtractive hybridization libraries of testis vs. inactive gonad. We obtained 352 and 177 ESTs by clone sequencing, and using pyrosequencing (454-Roche) we maximized the identified ESTs to 34,276 reads. A total of 1,153 genes from the testis library had a blastx hit and GO annotation, including genes specific for meiosis, spermatogenesis, sex-differentiation, and transposable elements. Some of the identified meiosis genes function in chromosome pairing (*scp2*, *scp3*), recombination and DNA repair (*dmc1*, *rad51*, *ccnb1ip1*/*hei10*), and meiotic checkpoints (*rad1*, *hormad1*, *dtl*/*cdt2*). Gene expression analyses in different gametogenic stages in both sexual regions of the gonad of meiosis genes confirmed that the expression was specific or increased towards the maturing testis. Spermatogenesis genes included known testis-specific ones (*kelch-10*, *shippo1*, *adad1*), with some of these known to be associated to sterility. Sex differentiation genes included one of the most conserved genes at the bottom of the sex-determination cascade (*dmrt1*). Transcript from transposable elements, reverse transcriptase, and transposases in this library evidenced that transposition is an active process during spermatogenesis in *N. subnodosus*. In relation to the inactive library, we identified 833 transcripts with functional annotation related to activation of the transcription and translation machinery, as well as to germline control and maintenance.

## Introduction

The Pacific lion-paw scallop *Nodipecten subnodosus* is a functional hermaphrodite Pectinidae species. It is considered the largest harvested scallop in the American continent, reaching a shell length of 16 cm. Anatomically, it is characterized by a clear and marked organ differentiation [1] in contrast to most non-pectinid bivalves, including the gonad that is a distinctly separated organ, and composed of ovary and testis parts (see Fig. 1 in [2]). Reproduction of *N. subnodosus* is discontinuous, with one major seasonal maturation peak per year [3], [4], with the first maturity occurring at a size above 5 cm shell length. After this size, both sexual parts develop synchronously, and spawning can occur by either sperm or egg release first [5]. The gametogenesis process occurs in the functional structure of the gonad, called acinus, in a similar pattern to other pectinid species, with gametes produced from primordial germinal cells located in the inner margin of the acinus. In the testis, sperm cells mature from germ cells in a radial process, and all the meiotic stages are represented in the acinus during gonad maturation, contrary to ovary, in which oocytes are arrested in metaphase-I until external fertilization [6]. In a transcriptional context, the testicular part of the gonad is ideal for the discovery of genes involved in the gametogenic process, as genes participating in mitosis of germ cells, meiosis and recombination, and post-meiotic or differentiation will be expressed [7]. Furthermore, sex determination/differentiation [8], [9] and transposable elements [10] are enriched or specifically expressed during testis differentiation and development. The discovery of reproductive genes in a Pectinidae species as *N. subnodosus* will offer the opportunity for further studies on the genetics of functional hermaphroditism, from germ cells maintenance to sex differentiation, as well as gamete differentiation through meiosis.

**Figure 1:**
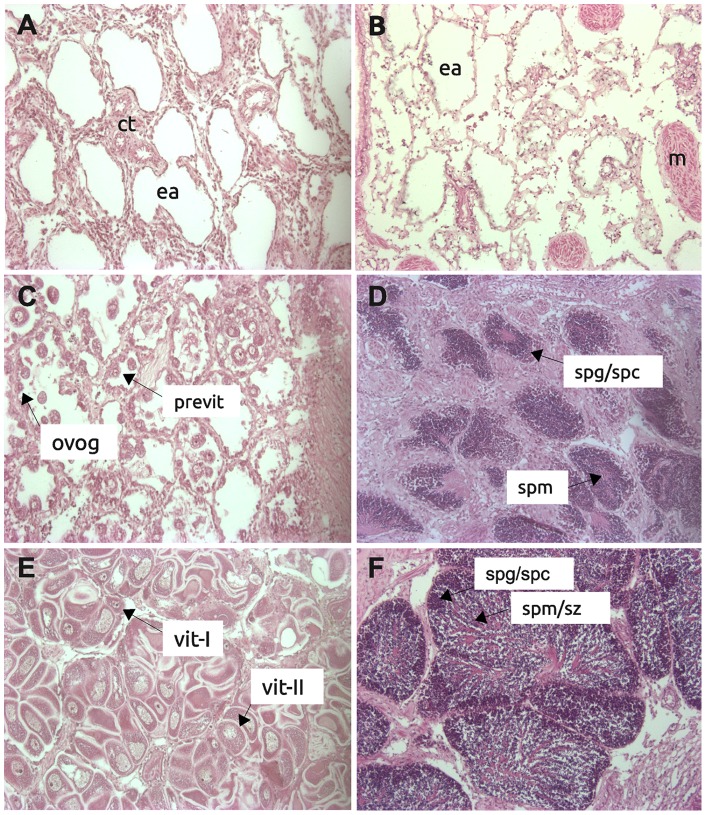
Reproductive stages evaluated in this study. Microphotographs of the reproductive stages evaluated in this study: inactive stage at pre-gametogenesis (A) and after spawning (B); early maturation of the ovary (C) and testis part (D); Late maturation of the ovary (E) and testis part (F). Tissues or cell types are denoted as follows: connective tissue (ct), empty acinus (ea), muscle fibers (m), oogonia (ovog), previtellogenic oocytes (previt), primary vitellogenic oocytes (vit-I),secondary vitellogenic oocytes (vit-II), spermatogonia (spg), spermatocytes (spc), spermatid (spm), spermatozoa (spz).

In *N. subnodosus*, biotechnological tools such as triploid production have been investigated for their applicability in aquaculture as a way to increase meat production and as a genetic confinement method to protect native populations when hatchery-produced scallops are grown within protected areas (JL Ramírez-Arce and AM Ibarra, unpublished). These studies have shown that the triploid condition induced by inhibiting the release of the second polar body in fertilized eggs, and therefore the conclusion of meiosis II, induces not only functional sterility, but also structural sterility as practically no gametes of either sex are produced in adult triploids [11]. This is not the case for all triploid mollusks, but complete or structural sterility has been found for some triploid-induced species [12]–[14] whereas other species, including other scallops, are capable of producing gametes [15]–[17], even if they are aneuploids [18]. However, it is not known if tolerance to aneuploidy causes the differences in triploid sterility between species in other taxa, and other mechanisms might be involved.

It has been suggested that sex chromosomes, when heterogametic chromosomes exist, might be a cause for the differences seen between triploid males and females in their ability to produce gametes [19], similarly to what has been proposed for sterility in hybrids [20], [21]. However, to our knowledge, sex chromosomes have not been described as yet for bivalves, although they have for gastropods [22]. Furthermore, for true hermaphrodites such as certain scallop species, heterogametic sex chromosomes are not expected even if sex chromosomes are present. A second cause for triploid sterility, at least in the plant kingdom, has been associated to methylation-dependent deregulation of genes or genomic sequences, such as transposons [23]. In the animal kingdom, despite the existence of mechanisms like DNA methylation and heterochromatin formation that prevent propagation and transposition; deregulation of transposable elements (TEs) during spermatogenesis is known to occur. An additional mechanism is also known to regulate TEs: small RNAs known as Piwi-interacting RNAs (piRNAs) [24]. Transposable element deregulation has not been studied in triploid animals although a recent study in shrimp found that a transposase, the enzyme associated to transposons and their movement, was qualitatively expressed at higher levels in the ovary of triploids compared to diploids [25]. A third possibility for triploid sterility is an induced arrest of gametogenesis at meiotic checkpoints. The sterility in triploid mollusks was proposed to be associated with a pachytene checkpoint after finding that gametogenic arrest occurred at the zygotene-pachytene stage of prophase I of meiosis in the scallop *Argopecten ventricosus* [26], [27]. Meiotic checkpoints function through genes that halt meiotic progression during DNA replication, recombination, or chromosome synapsis when a problem is detected in the early stages of meiosis [28]–[30], preventing the formation of aneuploid gametes. Checkpoint mechanisms have been found to function differently between sexes, as well as between species [31], with some species being more tolerant to aneuploidy. Interestingly, differences between triploid mollusk species in their degree of sterility can also be associated to their tolerance to aneuploidy. For example, the oyster *Crassostrea gigas* is only partially sterile when triploid and is known to be viable under aneuploid conditions, even though its growth can be affected [32]–[34].

To begin understanding the cause(s) of sterility in triploid scallop species, genomic and transcriptomic information associated with gametogenesis is necessary. One commonly used strategy to isolate tissue- or condition-specific genes involves the generation of suppression subtractive hybridization libraries –SSH– [35]. Testis-specific SSH libraries have been generated for a number of organisms, and have relied on cloning subtracted transcripts for sequencing by traditional Sanger capillary methods (e.g. [36]–[42]). In this work we report the results obtained by using a new approach for maximizing the number of tissue-specific transcripts at the lowest cost, especially for species with no or minimal genomic information: the generation of spermatogenesis transcriptomic information obtained after suppression subtractive hybridization of maturing testis and inactive gonad, combined with next-generation sequencing (NGS) by 454 GS FLX platform. Maturing testis and inactive gonad tissues were chosen to mainly focus on early male gametogenesis-expressed genes, sex differentiation and meiosis related genes. By using this approach, a large number of genes for which the annotation suggested their involvement in different steps of the spermatogenesis process, including meiosis and checkpoint genes, sex-differentiation genes, and germline control genes were isolated and annotated. Furthermore, a number of genes from transposable elements and microsatellite loci were also discovered. This work provides a panel of candidate transcripts to investigate the triploid-induced sterility in the scallop *N. subnodosus*.

## Materials and Methods

### Ethics statement

The lion's paw scallop is not considered as an endangered or protected species in any Mexican or international species catalog, including the CITES list (www.cites.org). Samples from Loreto Bay, a National Park, were collected with all the permits issued by the government agency CONANP in accordance to the funded project from the Federal Agencies CONACyT-SEMARNAT S0010-2006: “Biological model to reach a balance between conservation and sustainable use of marine resources in natural parks and reserves. Study case: lion's paw scallop (*Nodipecten subnodosus*)”. In the case of the animals collected from Magdalena Bay, those were obtained from a commercial culture site at Rancho Bueno Area, managed by the company “Cultemar S.A. de C.V.”, and the organisms were produced in our laboratory from broodstock collected in Laguna Ojo de Liebre, BCS Mexico, with all permits issued by the federal agency CONAPESCA.

### Biological material

For SSH libraries, hatchery produced scallops from three pooled half-sib families (eggs from one individual fertilized with sperm from three other individuals) were collected after grow-out in the field (Bahía de Loreto, Mexico) at approximately 5–6 cm shell length. A total of 45 scallop gonads were histologically examined, selecting 12 samples of testis in late maturing stage, as well as eleven samples from whole gonad tissue in post-spawned inactive stage, and used for RNA isolation. For gene expression analyses by qPCR, 88 scallops grown at Bahía Magdalena, Mexico, were sampled and also analyzed to determine their gametogenic stage. An anatomical description of the hermaphrodite gonad and how the testis and ovary were dissected can be found in Llera-Herrera et al. [2]. We grouped the reproductive stages as: inactive, early maturing testis or ovary, and late maturing testis or ovary (Fig. 1), which are those referred as undifferentiated, early development, and late development/ripe stages, respectively, in [43]. A total of 30 gonad samples were selected based on their gametogenic stage, conforming groups of six samples within each of the stages.

### Suppressive Subtractive Hybridization libraries

Approximately 50 mg from each sample within the two gametogenic stages (inactive and late maturing testis) were pooled and homogenized (Polytron PT 1300D, Kinematica, Switzerland) in 50 ml conical tubes, with ten volumes of TriPure reagent (Roche, Penzverg, Germany) per volume of tissues. Total RNAs were extracted following the manufacturer's protocol, UV-quantified, and analyzed for purity by spectrophotometry (NanoDrop-1000, Thermo, Walthman, MA). Quality was verified by agarose gel electrophoresis. Poly(A)-RNA was selected from each pool of total RNA with oligo-d(T) cellulose of the Poly-A-Pure kit (Ambion, Austin, TX) and using as starting material 1–2 mg of freshly prepared RNA pools, resuspended in 4 ml of 0.45 M NaCl, following the manufacturer's protocol. The final yield of the poly(A)-selected mRNA was analyzed by UV-spectrophotometry.

Subtractive libraries [35] were produced using the PCR-select cDNA subtraction kit (Clontech, Palo Alto, CA) following the protocol given by the manufacturer. 2 μg of poly(A) RNA coming from each pool (maturing testis and inactive gonad) were used for double-strand cDNA, *Rsa*I digestion. Then, restriction digests were separated as tester/driver to perform reciprocal hybridizations between conditions. In each library, tester-cDNA was ligated to adapters A and B separately, and then hybridized with five times the amount of driver cDNA to obtain cDNA fragments overrepresented in the tester samples. By swapping each biological condition as tester/driver we obtained cDNA fragments overrepresented in the corresponding contrasting condition. Each set of tester-cDNA hybridized with driver-cDNA was subjected to PCR amplification with PCR primer-1 (sequence given in the PCR-select cDNA subtraction kit of Clontech) and using Advantage cDNA polymerase mix (Clontech), as suggested by the standard protocol.

Two distinct strategies for the final preparation of the libraries and their sequencing were used: (a) the traditional cloning into competent *E. coli* cells for Sanger sequencing, and (b) an adapted technique for amplicon library sequencing by 454-FLX platform (Life Sciences; Branford, CT):

- Sanger sequencing: Nested PCR products with conventional primers (nested primer-1 and nested primer-2R; sequences given in the PCR-select cDNA subtraction kit, Clontech) were directly ligated into pGEM-T easy (Promega, Madison, WI) or TOPO pCR4.0 (Invitrogen, Carlsbad, CA) and used for transformation into *E. coli* DH-5α competent cells by electroporation. 564 individual clones (376 from the maturing testis library and 188 from the inactive gonad library) selected in x-gal/ampicillin LB agar plates were picked-up and deposited into sterile ELISA 96-well agar plates for single pass sequencing by capillary electrophoresis direct from bacterial clones using BigDye terminator and the T7 universal sequencing primer (Genewiz, NJ).
- 454-Roche sequencing: modified sets of primers were used for the nested PCR in the last step of SSH library protocol; those primers started in the 5'-end with the 454-Roche adapter sequence for amplicon library protocol, followed by an internal MID barcode, and finally the sequence of the original nested primer (Clontech SSH kit) in the 3'-end (recommendations given on the technical manual for unidirectional sequencing of Lib-L amplicon libraries; App No 001-2009, Roche Applied Science, Indianapolis, IN). To identify those reads coming from each library, MID7 and MID8 barcodes were used for the late maturing testis and inactive gonad libraries, respectively. Once the PCR were performed, we generated libraries ready to be sequenced by 454-Roche technology (454 Life Sciences, IN) by the amplicon library protocol of 454. Sequencing was done in one direction, with the Lib-L emPCR FLX chemistry and using an equivalent of a 1/8-plate by, at the GenoSeq Core Facility at UCLA, CA.

### Bioinformatics workflow

Sanger trace reads from SSH libraries clones were processed in batch for base-calling using Phred [44] (kindly provided by Phil Green, University of Washington) under default parameters and trimming all sequence regions with a Phred quality score lower than 20. For the 454-FLX sequencing of SSH libraries, trace files (*.SFF) were first separated according to their MID adapters, and base-calling was performed by means of script packages *sfffile* and *sffinfo,* included in the Newbler software (454 Life Sciences). Sanger reads were deposited into dbEST-NCBI database (Genbank accessions JZ199499 to JZ200027), and the 454-FLX reads were uploaded through the European Nucleotide Archive Study ERP002162 (ENA/SRA-NCBI accessions ERX199134 and ERX199135). A hybrid assembly was conducted including the reads obtained from clone sequencing by Sanger and amplicon libraries from 454-FLX sequencing using the Newbler GS *de novo* Assembler ver. 2.6 (454 Life Sciences), with the cDNA mode option, a minimum overlap of 30 bases and 90% similarity, and with the rest of the computational parameters set to default values.

The final dataset of isotigs (putative distinct transcripts; an equivalent term to the widely used “contig” given by Newbler software) and singletons (single reads that were not included into isotigs after assembling) were annotated using the Blast2GO tool, a platform-independent suite for BLAST-search description annotation, gene ontology (GO) mapping and data management [45]. BLASTX searches were conducted through the NCBI server, considering an E-value cutoff of 1×10^−3^, after the release of the annotated protein sequences of *Crassostrea gigas* genome sequencing project (NCBI BioProject PRHNA70283; [46] into the Genbank-NCBI non-redundant database. GO mapping was done after the blastx search, and it included a final integration of the relational GO categories with the Annex option. The uniqueness of sequences per library was obtained from the final assembly using a program written in Perl that identifies isotigs resulting from sequences in both libraries. An enrichment analysis of ontology terms was performed for the isotigs and singletons that were represented in each library, according to a Fisher-exact test in the Blast2GO software. Transcripts from the maturing testis library were extracted from the total pool of transcripts, searching the descriptions and GO terms with the purpose of summarizing genes by the following functional categories: meiosis, spermatogenesis, sex- and transposon-related.

### Gene expression analyses for meiotic transcripts

Relative quantification of gene expression was performed on identified meiosis-related genes by qPCR on undifferentiated gonads, testis and ovary gonads, including both early and late maturation stages (Fig. 1). For each of these stages, six individual tissues were selected. Samples of individual tissues were processed in parallel for RNA isolation, DNAse treatment and cDNA synthesis, as detailed in [2]. For qPCR analysis of gene expression, primer pairs were designed for each gene (Table S1). The two reference genes used were chosen according to their stability assessed in a previous study on the same samples used in this work: *ribosomal protein L8* (Genbank access JN034908.1), and *ribosomal RNA 18s* subunit ([2]).

qPCR reactions were done in duplicate on a 96-well thermal cycler (CFX-96, Bio Rad, Hercules CA) using a master mix containing 2.5 mM MgCl_2_, 200 μM dNTP (each), 0.45 U of Go*Taq* DNA polymerase (Promega), optimized concentration of each pair of primers, ranging from 0.3 to 0.5 μM, and 1x EvaGreen fluorescent dye (Biotium, CA) in 15 μl of final volume per reaction [2]. PCR conditions were: 94°C 3 min; 40 cycles of 94°C (10 s), 60°C (15 s) and 72°C (30 s), acquiring the fluorescence at 77°C (2 s). At the end, a dissociation step from 60°C to 94°C (1°C/s) was performed, and the resulting melting curve obtained for each reaction was inspected for specificity. For each gene, amplification efficiencies (E) were determined by the slope calculation of 5-fold serial dilutions of cDNA. Efficiency values were then obtained from the slope of the log-linear function of the dilution factor *vs*. fluorescence, using the equation E =  10(−1/slope) [47], and used for gene-specific efficiency correction in the relative quantification model as recommended by [48]. Relative quantities for each, target and reference genes, were estimated with the equation RQ  =  (1+E)^(Cq *mean* – Cq).^ Relative expression values (RE) were calculated from the ratio of relative quantity (RQ) of each individual sample with the equation RE  =  RQ_t_/RQ_nf_ (t = target gene and nf  =  normalization factor  =  geometric mean of reference genes). Cq values above 35 were considered as no amplification. Statistical analyses were performed by one-way ANOVA after transforming RE to natural logarithms. Means were compared using Fisher's post-hoc test, using Statistica version 7 software (StatSoft; Tulsa, OK). Results are presented transformed back from the natural logarithms.

### Microsatellite identification

Microsatellites – single sequence repeats (SSR) – were screened *in silico* within the original unassembled reads using QDD2 software [49]. It is implemented in Perl language and uses ClustalW2 [50] and blast+ [51] to align the reads in flanking regions and thus reducing redundancy of potential microsatellites, and Primer3 v1.1.4 [52] was used for designing the best pair of primers for each microsatellite locus in the flanking regions. The minimum number of repetitions allowed was five for di-, tri-, tetra-, penta- and hexanucleotide repeats. This software was also used to determine those SSR that were polymorphic based on the variations observed on the single reads. Microsatellites with flanking regions, with the repeated motif masked out, were screened by blastn against the nr database of Genbank.

## Results

### Subtractive library sequencing, assembly and annotation

Combining Sanger and 454-FLX sequences, we obtained a total of 34,276 valid reads of at least 50 bp with a quality score over Q20. The number of Sanger reads meeting these quality criteria were 352 and 177 from the maturing testis gonad and inactive gonad libraries, respectively. With 454-FLX sequencing we obtained 19,876 and 13,857 reads, respectively. A hybrid assembly of all reads with the Newbler software resulted in 3,727 isotigs and 8,279 singletons. The N50 isotig size was 429 bp. We found 2,037 sequences (isotigs or singletons) with at least one significant blastx hit corresponding to 16.9%; and within those, 51% of the top-blast hits were against proteins of *Crassostrea gigas*, a bivalve mollusk species with a recently sequenced genome [46]. With regard to each specific library, 1,153 transcripts from the late maturing testis and 833 from the inactive gonad library had a significant blast hit against the *nr* Genbank database (Table S2), and from those numbers, 768 (67%) and 697 (84%) respectively, had at least one Gene Ontology term significantly associated by blast2go (Table S3).

### Functional annotation

Subtraction efficiency was evidenced by finding that only 91 isotigs (from a total of 3,727) were built from both libraries. From those, 48 had at least one blastx hit and at least one significantly associated GO-term, and 3 had a blastx hit but no known annotation. Among the transcripts with annotation, there were different ribosomal proteins (n = 13), cytochrome synthase (n = 4), ATP synthases (n = 3), and NADH dehydrogenases (n = 2), among others. The low relative abundance of these highly and generally expressed genes further supports the success of the subtraction process. The remaining 43 transcripts had no blastx hit or annotation. Further proof of the subtraction efficiency for identifying genes specific to the biological conditions being compared was obtained with an enrichment analysis of ontology terms. A comparison between isotigs and singletons from each library (Table S4) showed significant GO terms that are specifically enriched in either the maturing testis or the inactive gonad library.

**Figure 2:**
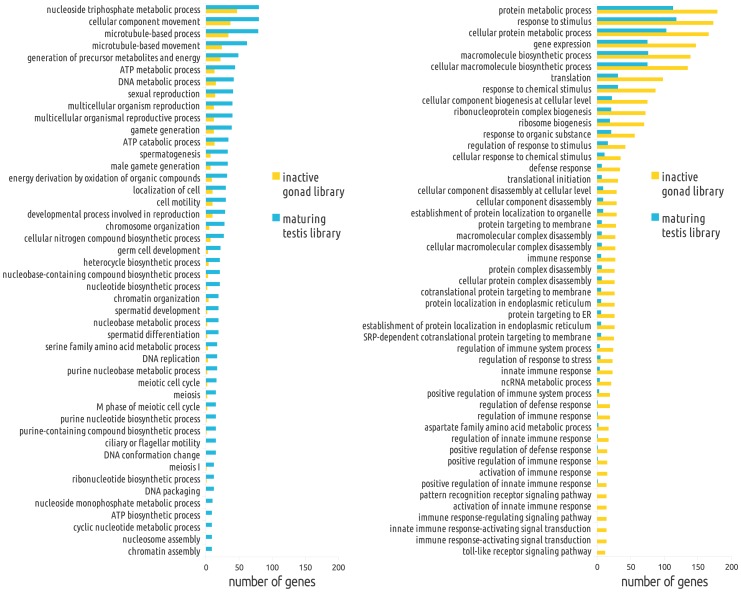
Functional ontology terms overrepresented in each library. Enrichment analysis of functional ontology terms between reciprocally subtracted libraries, and overrepresented in the maturing testis library (left) or in the inactive library (right).

Terms corresponding to Meiosis (GO:0007126), Meiosis-I (GO:0007127), meiotic cell cycle (GO:0051321) and M phase of meiotic cell cycle (GO:0051327) were highly associated to genes found in the male gonad library (Fig. 2). Additionally, terms related to spermatogenesis were also found to be enriched in this library. Among the identified genes in the maturing testis library, several meiotic genes involved in DNA replication fidelity, chromosome synapsis, recombination, and meiotic checkpoint (Table 1) were also found. Genes identified within spermatogenesis (Table 2) code for sperm protein antigens, testis-specific protein kinases, and several constituents of the flagella, among others. In addition to meiosis and spermatogenesis, we identified genes with the word ‘sex’ in their descriptions (Table 2), such as *dmrt1* (*doublesex and mab-3 related transcription factor 1*), *dual specificity protein kinase* (*clk2*), and *kelch-10 like*. Finally, we identified transcripts annotated with the prefix ‘transpos’ or ‘retrotranspos’ for transposable elements and retrotransposons (Table 3), including among others, piggyBac transposable element-derived protein 3-like, retrotransposon transcriptase, RNA-directed DNA polymerase derived from two transposable elements, Bs and *jockey*-like, and retrovirus-related pol polyprotein from type-1 retrotransposable element R2.

**Table 1:** Meiosis-associated genes expressed in the maturing testis library, with GO terms related to meiosis/meiotic, recombination, repair, checkpoint.

Annotation description	Best e-value	Best hit
ATP-dependent DNA helicase Q1	1.27 e-45	EKC18968
ATP-dependent DNA helicase Q4	1.14 e-21	EKC33210
brain tumor protein	2.77 e-15	EGI57493
cell cycle checkpoint protein RAD1	5.58 e-22	BAD86790
cyclin dependent kinase 1	2.10 e-10	ADZ23998
denticleless protein homolog	2.99 e-36	EKC37484
citrate synthase	2.53 e-49	EDS33990.1
DNA ligase 1	1.09 e-16	XP_003127315
	4.63 e-47	EKC32253
	7.63 e-40	XP_002160108
	4.03 e-34	EFN72821
DNA polymerase delta subunit 2	3.29 e-60	EKC39895
DNA repair protein RAD51 homolog 3	1.06 e-62	EKC25786
DNA replication licensing factor MCM6	2.91 e-15	BAG61268
dual specificity protein kinase TTK	1.59 e38	EKC17444
e3 ubiquitin-protein ligase CCNB1IP1	9.79 e-19	XP_780975
	1.28 e-36	XP_002588845
histone 3	2.92 e-57	CAJ81662
histone 3.2-like	2.48 e-18	EJT46200
HORMA domain-containing protein 1-like	8.50 e-85	XP_002168952
	3.82 e-14	XP_003727321
importin subunit alpha-2	6.81 e-20	EKC18904
integrase family protein	4.29 e-22	XP_003390809
	3.38 e-14	EKC22946
	3.17 e-8	EKC28263
meiotic recombination protein DMC1/LIM15 homolog	1.89 e-94	XP_003221056
	5.91 e-45	XP_002831176
	5.66 e-8	XP_001367929
p33 RINGO	4.49 e-48	EKC24735
RuvB-like protein 2	3.26 e-63	EKC28232
serine threonine-protein kinase PLK1	1.18 e-48	EKC29729
synaptonemal complex central element protein 2	4.81 e-11	XP_002591412
synaptonemal complex protein 3	1.32 e-45	EKC40091
TFIIH basal transcription factor complex helicase subunit	1.84 e-11	AEO89688
ubiquitin-conjugating enzyme e2 c isoform 2	7.40 e-6	NP_861515
Werner syndrome ATP-dependent helicase	2.10 e-11	XP_003389810
zinc finger and BTB domain-containing protein 40	1.48 e-13	XP_002735651

**Table 2:** Genes expressed in the maturing testis library extracted after searching GO terms with the prefixes sperm/spermatogenesis/spermatid and the term sex.

Annotation description	Best e-value	Best hit
adenosine deaminase domain-containing protein 1-like isoform 2	1.14 e-10	EKC39025
C-Myc binding protein	7.84 e-44	XP_002604299
coiled-coil domain-containing protein 135	1.57 e-23	EKC19048
DnaJ subfamily member 13	5.88 e-51	EKC20056
doublesex and mab-3 related transcription factor 1	4.69 e-22	CAC42778
dual specificity protein kinase	1.86 e-7	XP_002432301
dynein heavy chain	5.06 e-90	EKC20777
growth arrest-specific protein 8	6.30 e-64	EKC37009
H1 histone member oocyte-specific-like	3.75 e-21	P22974
Kelch-like protein 10	1.92 e-57	EKC42522
	2.72 e-32	EFN76175
motile sperm domain-containing protein 2	1.65 e-26	EKC28345
nucleoside diphosphate kinase homolog 5-like	8.83 e-38	EKC43147
outer dense fiber protein 3-like protein 2-like	1.25 e-16	XP_002732801
parkin coregulated gene/protein homolog	2.68 e-57	EKC36429
	8.50 e-54	XP_003215805
peptidyl-prolyl cis-trans isomerase FKBP4	2.31 e-44	EKC41424
predicted protein (DM domain)	7.00 e-6	XP_001632590
probable ubiquitin carboxyl-terminal hydrolase FAF-X	4.12 e-11	XP_001627156
ropporin-1-like protein	2.73 e-126	EKC31617
sperm flagellar protein 2	1.07 e-7	XP_001623400
sperm phosphodiesterase 5	1.81 e-34	EKC21406
sperm-associated antigen 16 protein	2.32 e-81	EKC18115
	1.16 e-14	XP_002591791
sperm-associated antigen 17	2.31 e-54	EKC26001
spermatogenesis associated 4	1.66 e-83	EKC20206
spermatogenesis-associated protein 1	5.44 e-16	XP_002733667
tektin 2	7.57 e-73	EKC18085
	1.06 e-14	XP_002591791
testis-specific serine threonine-protein kinase 1	5.53 e-31	XP_002738751
testis-specific serine threonine-protein kinase 2	1.52 e-17	EHA99087
testis-specific serine threonine-protein kinase 3-like	9.62 e-24	EKC40940

**Table 3:** Genes expressed in the maturing testis library extracted after searching GO terms for transposable, transposase, retrotransposon, retrotransposable and DNA-polymerase.

Annotation description	Best e-value	Best hit
DNA-mediated transposase	1.14 e-13	ABH09251
piggyBac transposable element-derived protein 3-like	4.71 e-6	XP_003706354
tyrosine recombinase-like	6.04 e-9	DAA01995
pol-like polymerase (25 transcripts)	1.27 e-30	XP_002160589
retrotransposon transcriptase	4.82 e-10	EFN57005
retrotransposon unclassified	5.10 e-25	BAC82624
retrotransposon-like family member (*retr-1*)-like	7.34 e-41	XP_003730886
retrovirus-related Pol polyprotein from type-1 retrotransposable element R2	2.51 e-40	XP_002127101
RNA-directed DNA polymerase from transposon Bs (13 transcripts)	2.89 e-21	XP_002127101
RNA-directed DNA polymerase from mobile element *jockey*-like (20 transcripts)	7.50 e-27	XP003727887
transposable element TCB2 transposase	1.28 e-5	ABV31711

In contrast to the maturing testis, in the inactive gonad library many of the GO terms were associated to translation machinery and ribosome biosynthesis, as well as immune and stress response (Fig. 2). However, other interesting genes were also identified from the inactive gonad: genes involved in sex differentiation and germline control and maintenance, such as *notch homolog 2*, *btg member 2*, *pumilio domain-containing protein*, *cleavage and polyadenylation specificity factor subunit 5*, *nuclear receptor subfamily 0 group b member 1*, *transcription factor btf3 homolog 4*, *ccr4-not transcription complex subunit 10*, and *transcription factor hes-1,* among others (Tables S2 and S3).

### Expression analysis of meiosis genes

**Figure 3:**
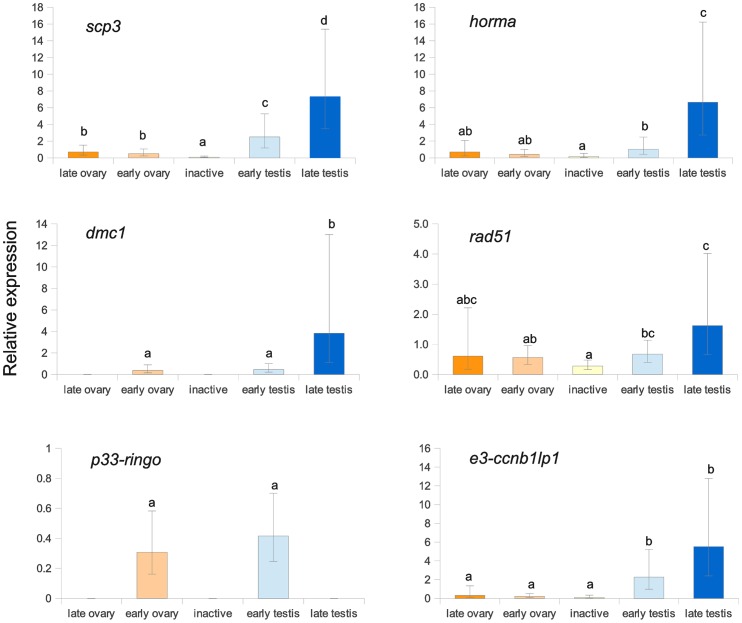
Relative gene expression from the maturing testis library transcripts. Relative expression of selected genes from the maturing testis library over different gametogenic conditions (n = 6 individuals for each stage): late and early oogenesis on the female part, inactive gonad, and early and late maturing testis part. Different letters denote statistical significant differences (*P*<0.05; Fisher post-hoc test for differences between groups).

As one of our main objectives on this work was to identify meiosis genes for later research on understanding triploids sterility, we next examined the expression of six identified meiosis-related genes by qPCR, across testis and ovary in both early and late maturation stages, as well as undifferentiated gonads (Fig. 3). Five of the six genes showed an increase in expression profile from inactive through the maturation stages of the gonad, and three of those showed significant increases in expression for testis: *HORMA-domain containing protein 1*, *scp3 (synaptonemal complex protein 3)*, and *dmc1* (Fig 3). Although some expression for *e3 ubiquitin ligase ccnb1lp1* was seen in the ovary stages, it did not show significant differences with the inactive gonad, whereas in the testis the expression of this gene was significantly higher than in other conditions, and showed a tendency to increase from early to late maturation stages. Expression of *rad51* was observed in all gonad stages/sexual parts, but was also increased in the late maturing testis stage compared to other stages. The only gene that did not show significantly higher expression in late testis or ovary was *p33-RINGO.* This gene was only expressed in the early maturing gonad.

### Identified microsatellites

We identified 507 sequences with potential microsatellite loci, and successfully designed PCR primers for 310 of them. From these, a total of 272 pure and 38 compound microsatellites were found. A table of the microsatellite sequences, designed primers and the best-hit on Genbank is presented in Table S5. One microsatellite found in a singleton sequence (ENA accession ERR224474.16682) perfectly matched with the NsubA1B12 microsatellite from Genbank (accession no. FJ986341.1).

## Discussion

### SSH generation and sequencing by 454-Roche FLX chemistry

Subtractive hybridization library generation and sequencing has been a widely used technique for obtaining transcriptomic information of genes that are specifically expressed under contrasting biological conditions, including testis vs. ovary or testis developmental stages in marine vertebrates and invertebrates [41], [42], [53], [54]. Because it is based on *de novo* sequencing, it is especially apt for non-model organisms for which there is no genomic information, and because of its sensitivity, it allows the isolation of rare, low-copy number transcripts [55]. However, one disadvantage might be that the sequencing effort and cost required to obtain enough information of condition-specific genes can be high since thousands of individually picked clones have to be obtained, making this method very expensive compared to the recently emerged next generation sequencing technologies. We have shown that by combining both, subtractive hybridization and massive sequencing technologies, it is possible to obtain a large amount of *de novo* functional information of differentially expressed transcripts in the maturing testis *versus* the inactive gonad of the lion's paw scallop: we successfully obtained on the order of 10 to 100 times more sequences than previous works analyzing testis genes [41], [42], [53]. A substantial sequencing effort using SSH libraries to generate *de novo* transcriptomic information was done initially for the Pacific oyster *C. gigas*, sequencing 8,064 clones from 6 reciprocally subtracted libraries of different organs [56]. Until now, we have only found two works using SSH with next generation sequencing platforms, one with a toxic dinoflagellate and the other with soybean [57]–[59]. Our results suggest that the strategy of combining SSH libraries and massive sequencing could increase the amount of transcriptomic information for very rare genes in comparative studies of non-model marine organisms.

The number of isotigs that were assembled with reads coming from the two libraries comprised only 2.6% of a total of 3,727. Previous publications [55], [60] reported that SSH libraries are normally enriched with very rare transcripts, but that very abundant transcripts in both tester and driver can escape from subtractive normalization. These transcripts include ribosomal proteins -constituents of the ribosomes- that are highly transcribed and are also implicated in many extraribosomal functions in addition to the translation machinery [61]. Similarly, some transcripts such as cytochrome C oxidase, ATP synthases, and NADH dehydrogenases, which function in mitochondria, and participate in basal energetic demands, should be expressed in both maturing testis and inactive gonad. Two more interesting transcripts were also found in both libraries. One of these encodes for the protein MAGO NASHI homolog, which in *Drosophila melanogaster* is involved in establishing the delineation of the anteroposterior axis in embryos through proper localization of *oskar* mRNA to the posterior pole and the subsequent body axis determination [62]. In Caenorhabditis *elegans*, a hermaphrodite species such as the one being studied, this gene is required for embryonic morphogenesis like in *Drosophila*, but also for the regulation of the sperm to oocyte switch in adult hermaphrodites. This was first demonstrated by RNAi studies in which knockdown worms showed masculinization of the germline [63]. These authors proposed that *mago nashi* inhibits masculinizing genes like *fog*, *fem*, and *gld*, allowing for oogenesis to occur. This gene has been shown to form part of the exon-exon junction complex of spliced mRNA, but its mode of action on promoting the sperm to oocyte switch remains unknown [64]. More recent studies have shown that MAGO NASHI protein might also be involved in altering the splicing pattern of specific genes in *Drosophila* outside the germline [65].

The second transcript, annotated as *protein yellow*, forms part of the YELLOW family of proteins, until now only identified in Arthropods, fungi, and some bacteria [66], that are known to be involved in mating behavior and melanization, and have also been reported as overexpressed by gene amplification in follicle cells of *Drosophila's* ovary, where it is needed for proper eggshell formation [67]. YELLOW proteins have been found associated with male-specific FRUITLESS protein in neurons of the central nervous system (CNS), but they are also present in females CNS [68]. Whereas the specific function of this gene in the scallop gonad is not known, the finding of this transcript in the inactive gonad and the maturing testis is not unique to the scallop, since all YELLOW proteins have been found transcribed in multiple tissues in *Bombyx mori*, including both testis and ovary [69].

### Meiosis, recombination, and DNA repair genes found in the maturing testis gonad

Meiotic recombination is a process by which genetic crossing-over or exchange of homologous chromosomes occurs during gametogenesis. In some species, recombination depends on gender; for example, recombination is absent in *Drosophila melanogaster* males [70]. When recombination does not occur, transcription of genes coding for specific roles in recombination does not occur [71]. In the lion's paw scallop, meiotic recombination during male gamete production is known to occur, as it has been recently found that recombination rates for female and male microsatellite markers were similar [72]. Genes involved in meiotic recombination identified in our SSH libraries represent functional corroboration that recombination occurs during male meiosis in this species. For example, genes involved in recombination and synaptonemal complex (SC) formation were found in the maturing testis library. The synaptonemal complex is a meiosis-specific structure consisting of two parallel axial elements (AEs), each associated to a pair of sister chromatids, and a transversal filament located between the synapsed homologous chromosomes. Two genes coding for synaptonemal complex proteins, *scp2* and *scp3*, were found expressed in the maturing testis library. SCP2 and SCP3 are two of the three proteins that form the axial filaments of the synaptonemal complex in meiosis-I, and are essential for chromosome pairing since mutations in mice lead to absence of AE and chromosome synapsis [73]. Another protein participating in SC formation is HORMA-domain containing protein 1 (HORMAD1). This protein co-localizes with the SCPs in the axial element, and is a critical component of the synaptonemal complex. Mutant genotypes result in disruption of the localization of SCP2 and SCP3 during early recombination, with loss of the tripartite structure of the synaptonemal complex (axial and central), resulting in aneuploidy [74]. Our results for quantitative expression of *scp3* and *HORMAD1*, which increased from early to late testis maturation stage, indicate that synaptonemal complex formation, and therefore meiosis I, was occurring up to what we named ‘late maturing testing stage’. In fact, the main difference between the early and late testis stages was the size of the acini, with early testis having small acini and late testis large acini filled with all spermatogenic stages, including early spermatocyte stages (Fig 1). The expression of these genes during meiosis I is interesting, since morphological identification of primary and secondary spermatocytes has not been observed in this scallop. These genes might serve as future markers for this meiotic stage and this should be tested, similarly to what was done by Bannister et al. [75].

In addition to proper cohesion of homologous chromosomes during SC formation in meiosis, DNA must be repaired after the double-strand breaks required for recombination to occur. The third gene whose expression increased from early to late maturing testis was *dmc1*, coding for one of the two recombinases involved in the crucial step of repairing those breaks, DMC1 and RAD51. Although these proteins share a high level of sequence and structural similarity, they are known to have biochemical and functional differences. DMC1 is expressed exclusively in meiosis and is required for normal homolog pairing and SC formation [76]. On the other hand, RAD51, the eukaryotic homologue of the bacterial strand transfer enzyme RecA [77], is expressed in both mitosis and meiosis. DMC1 has higher affinity for binding ssDNA and RAD51 forms helical nucleoprotein filaments that promote strand invasion and formation of heteroduplex DNA [78]. These two proteins co-localize in recombination spots in an equimolar ratio during pachytene, and because both have the capability for protein interaction, it has been suggested that they form a functional complex with recombinase activity [77]. In the scallop, *dmc1* expression was restricted to those stages in both testis and ovary where meiosis I is expected to occur, confirming its specific recombination role in this species. In contrast, *rad51* expression was present in all gonad stages, including the inactive gonad, confirming that this gene is required in other DNA-repair processes besides meiosis. Currently, we aim to conduct further analyses of full-length structure and expression analyses for *scp3*, *dmc1*, and *HORMA domain containing protein 1* to establish their possible use as transcriptional markers of pachytene meiotic arrest in triploid scallops.

Another gene annotated as a meiosis gene that was found overexpressed in the testis gonad was *e3 ubiquitin ligase ccnb1ip1* (also known as *Human Enhancer of Invasion 10* -*Hei10*), a RING domain containing protein known to be essential for fertility in mice that participates in chiasmata formation by resolution of recombination intermediates [79]. This component of the ubiquitination machinery also participates in meiotic maturation at the meiotic prophase I-metaphase I boundary by regulating the balance in accumulation/degradation of cyclin B [80]. Although previously thought to have no orthologs in other organisms, it has been recently described in rice, and proposed to be a homolog of budding yeast ZIP3 and *Caenorhabditis elegans* ZHP-3 with a function in normal crossover formation during recombination [81]. In contrast to *HORMA domain containing protein 1* and *scp3*, the expression of *e3 ubiquitin ligase ccnb1ip1* did not increase from early to late maturing testis, which might be an indication of its main role being early in meiosis, during crossover formation. Furthermore, the expression pattern we observed for *e3 ubiquitin ligase ccnb1ip1* was specific to the testis, with expression in ovary not being different from the inactive gonad, suggesting that this gene is testis-specific in the scallop.

Among the genes annotated as participating in meiosis and analyzed for their expression between gonad stages and sex, *p33-ringo* was the only one with specific expression in early maturing stages of both ovary and testis. This gene, also known as *speedy*, was first implicated as an initiator of oocyte maturation by inducing the G2/M transition in *Xenopus* oocytes (Ferby et al. 1999, cited by [82]). It was later found to also induce G1/S phase progression when overexpressed in different human tissues, and more recently has been associated with promotion of tumors, possibly because of its capacity to prevent activation of checkpoints [83]. The fact that we did not detect any expression in maturing testis might be the result of this gene being under-expressed and not detectable by qPCR even if the subtraction was successful in isolating two identical reads that comprise the *p33-ringo* isotig. Furthermore, no expression in the maturing ovary indicates that this gene is not involved in oocyte maturation like in *Xenopus*. A recent study of the SPEEDY/RINGO family of proteins in vertebrates has found that this gene family is predominantly expressed in testis, although lower expression was detected in other tissues [84]. Future work after characterizing the full-length transcript of this gene will be important to understand its function in early meiosis in this scallop, especially when triploids are faced with a meiotic arrest.

Whereas several other proteins that are involved in chromosome synapsis and recombination were expressed in the scallop maturing testis, it is important to also mention other genes that are potentially involved in the timing and revision of these events in a cell-cycle context. Particularly, “checkpoint genes” (also included in Table 1) are promising members to be further analyzed in the context of triploid-induced sterility. A cell cycle checkpoint operates to maintain the proper integrity of the genome by ensuring the sequence of events that occurs during the process of cell division and genomic segregation [85]. In diverse species such as *S. cereviseae*, *C. elegans* and *D. melanogaster*, meiotic cells with defects in recombination and synapsis trigger a delay or arrest in the pachytene stage of prophase I (pachytene checkpoint) [21]. One of those meiotic checkpoint genes is *HORMA domain-containing protein 1*, already discussed as a gene participating in SC formation, but known to simultaneously direct several meiotic processes. In *C. elegans*, HTP-1, the homolog of HORMA D1, participates in the control of meiosis by inhibiting the assembly of SC between non-homologous chromosomes [86], triggering a ‘wait’ signal for synapsis progression until homology has been verified [30]. Another meiotic checkpoint gene, *denticleless protein homolog* (*denticleless E3 ubiquitin protein ligase homolog*), or *cdt2* in yeast, is known to control re-replication during the S-phase by promoting degradation of a protein required for pre-replication complex formation: CDT1 (chromatin licensing and DNA replication factor1). But it is also known as an essential component of the G2/M checkpoint in vertebrates [87], in agreement with this gene being overexpressed in the developing gonads of *Drosophila* [88].

Additionally, a dual role for certain proteins in both DNA damage-induced mitotic and meiotic checkpoint control mechanisms [89] is known to exist. We found at least two transcripts of this type in the maturing testis library. Rad1 is a checkpoint protein involved in arresting cell in G2 until DNA damage is repaired [90]. In humans, Rad1 protein is abundant in testis, and is associated with both synapsed and unsynapsed chromosomes during meiotic prophase I of spermatogenesis [89]. Another checkpoint gene found in the maturing testis was the *dual specificity protein kinase TTK*, reported as a checkpoint-activity protein that activates p53 by phosphorylation when the mitotic spindle is disrupted after a prolonged mitotic exit without cell division. In response to stress, activated p53 modulates the expression of target genes like p21, which are required for cell cycle arrest [91].

### Sex differentiation and testis -spermatogenesis- specific genes in the maturing testis

In organisms with chromosomal sex determination, once the sexual fate of the gonad has been established, sex-differentiation-specific genes are transcribed to establish the sexual fate of the gonad [92]. In functional hermaphrodites like the lion-paw scallop, sex differentiation genes are expected to lead to formation of the separated gonad-sex regions, with sex specific regulation in each region. Evidence for this is the discovery of several genes implicated in sex differentiation in the maturing testing library of the scallop. One of those genes was annotated as *dmrt1* (*doublesex and mab-3 related transcription factor 1*), known to regulate male sexual differentiation from nematodes to mammals. The *Drosophila* homolog of *dmrt* (*dsx*) is located at the bottom of the sex-determination cascade, and is one of the best characterized candidates for sex-differentiation mechanisms so far, in which sex-specific alternative splicing is finely regulated (reviewed by [93]). In *C. elegans*, the *mab-3* homolog is required also for sex-differentiation of somatic organs and sexual fates between hermaphrodites and males, and its sex-specific splicing is regulated by TRA-1 [94]. We are currently working on the characterization of the transcript and its protein product, and analyzing its expression in the context of sexual regions of the hermaphrodite gonad of *N. subnodosus.* Another gene annotated with a function in sex differentiation was *dual specificity protein kinase*, *clk-2*, whose protein CLK2 is known to prevent the use of exons 2 and 3 in the human homolog of *Drosophila* TRA-2, TRA2-BETA 1, a SR-like protein that regulates alternative splicing [95]. In *Drosophila*, mutations in the homolog of *clk* (*doa*) result in disruption of sex-specific splicing of *doublesex* pre-mRNA, whose splicing is known to depend on TRA and TRA-2 [96]. Finally, the last gene annotated as involved in sex differentiation was *fkbp52* (*peptidyl-prolyl cis-trans isomerase*), which codes for a co-chaperone for HSP90, and is known to be critical for proper development and differentiation of male reproductive organs in mammals [97].

In the hermaphrodite nematode *C. elegans*, testis-specific transcriptomic profiles have been established in comparison to the soma [98]. Testis-specific genes can be expressed by testis-specific promoters [99], [100] or by sex-specific splicing [101]. The set of testis-specific genes discovered in *N. subnodosus* (Table 2) includes some of the genes that are expressed specifically in the testis in other species. For example, *Kelch-like protein 10* was found expressed in mouse spermatids by *in situ* hybridization, and a search in the UniGene database indicated that most of the reported ESTs (45 of 46) for this gene were obtained from testis libraries [102]. Other sperm proteins, annotated as *sperm antigen 16* and *17* were found. Although they are simply annotated as ‘sperm antigens’ because they were initially discovered as immunoreactive proteins in experiments of target proteins for contraception in humans [103], they are known to be structural components of the sperm cells. For example, sperm antigen 16 (SPAG16), expressed in the diploid meiotic stage, locates in the central apparatus of the sperm tail and is essential for flagellar motility [104]. Another constituent of the sperm tail is the transcript identified as *outer dense fiber protein 3-like*, also known as SHIPPO1, is only expressed in haploid cells, and is a testis-specific protein located in the flagella of elongated spermatids and along the entire length of the mature sperm [105]. A protein known to interact with dsRNA, the adenosine deaminase domain-containing protein 1-like isoform 2 (*adad1*) was also found during spermatogenesis. This gene is testis specific, and in mice it is restricted to cells from the pachytene stage of spermatocyte until the spermatid stage [106]. Mutations in this gene are known to result in abnormal sperm and male sterility [107].

### Transposable elements and related enzymes discovered in the maturing testis library

Transposable Elements (TEs) are mobile elements that account for a large fraction of the genome in most eukaryotes, including mollusks, although the proportion of TEs has not been estimated for most species. In the recently released genome of the oyster *Crassostrea gigas*, it was estimated that miniature inverted-repeat transposable elements (MITEs) comprise 8.82% of the genome, and gene fragments derived from transposases and transcriptases were detected not only in the genome, but also as transcripts [46]. It is known that newly generated transposable elements have to be expressed in germ cells and their embryonic precursors to ensure their inheritance [10]. Relaxation of the epigenetic control for TE expression, together with integration by genome demethylation, leads to a potential disruptive incorporation of TEs into the genome [24], something that can be important for the sterility seen in triploids. The finding of diverse transposases and polymerases, such as DNA-mediated transposase, retrotransposon transcriptase and TCB2 transposases evidenced that, not only the transposon transcripts, but also some of the enzymes required for their retrotransposition and integration into the genome are actively expressed during spermatogenesis in the scallop. Among these TEs, we found a transcript for the *piggyBac transposable element-derived protein 3-like*, a short inverted-repeat-type DNA TE originally isolated from a moth genome (see [108]), and used as a vector for transgenesis in insects and parasitic protozoans [109], but that has not been previously reported in another mollusk.

Other TEs found expressed in the maturing testis were the retrotransposon-like *retr-1* and a second unclassified retrotransposon. Several transcripts of the reverse transcriptase of the *Jockey*-like element and transposon Bs were also found. These enzymes possess the transcriptase activity for the RNA-directed DNA polymerization required before the reintegration to the genome [110]. Surprisingly, given their evolutionary closeness and a large proportion of the oyster genome carrying TEs [46], none of the scallop TE transcripts produced a best hit with TEs from the oyster. Nevertheless, 11 proteins associated with TEs are predicted to be present in the genome of the oyster *Crassostrea gigas*, and two of those were also found in the scallop: an RNA-directed DNA polymerase from Bs and Tcb2 transposase. It will be interesting to evaluate if some of the newly discovered transcripts in the scallop are unregulated during the meiotic arrest in triploids.

### Genes found in the inactive gonad library

With regard to the inactive library, it is important to note that the samples used for library preparation were collected from the grow-out site in June, when the temperature is rising and chlorophyll concentration is decreasing. At the sampling date, 50% of the scallop population was in inactive reproductive phase, while the other 50% was maturing or mature, although the testis of some hermaphrodite scallops (10%) was already spawned (J.L. Ramirez-Arce, unpublished data). Histological observations of the sampled scallops indicated that some inactive organisms could, in some cases, surpass a first incomplete gametogenic process, with evidence of resorption of residual gametogenic cells given by the presence of hemocytes throughout the gonad tissue, both inside or surrounding the empty broken acini. This explains many of the transcripts found in the inactive gonad. The gene ontology enrichment analysis showed the functional categories that were differentially regulated between those reproductive conditions (inactive gonad *vs.* maturing testis). In contrast to the maturing testis library, in which meiosis, as well as spermatid and sperm development were overrepresented; in the inactive library immune and defense response, protein metabolism and ribonucleoprotein biogenesis were the overrepresented terms, suggesting an active protein turnover related to the regeneration of the acini in those post-spawned animals.

In addition, some genes were found in the inactive gonad that indicated other processes such as germline control and maintenance. Maintaining the germline after the first maturation and spawn would be expected in the scallop because a second maturation will occur approximately one year after the first one (see [4], [11]). Furthermore, the sexual identity of male or female germ cells has to be either maintained or specified when a new gametogenic cycle begins.

Among the genes found in the inactive gonad, *notch homolog 2*, a gene coding for the NOTCH receptor protein, is known to be responsible for keeping a balance between cell proliferation and apoptosis. NOTCH proteins are activated by interaction with specific ligands, and are then cleaved twice resulting in an intracellular cleaved NOTCH fragment that activates transcription of Notch target genes in the nucleus [111]. In *C. elegans*, GLP-1 is one of two Notch receptors, and GLP-1, together with FBF proteins, functions to promote continued mitotic divisions in the germline, inhibiting entry into meiosis [112]. FBF proteins 1 and 2 (**f**em-3 **b**inding **f**actors) named because their original discovery as a fem-3 binding protein [113], but now known to also control germline stem cells by repressing translation of two known regulators that promote meiosis [114], are homologs of PUMILIO or PUF proteins. PUF proteins are RNA binding proteins that are known to regulate translation of mRNAs by targeting regulatory elements in the 3'UTRs. Interestingly, a second transcript found in the inactive gonad was annotated as *pumilio domain-containing protein*. Whether the scallop germline uses a similar repression mechanism to that of the hermaphrodite nematode is not known, but it should be investigated in the future since other transcripts found in the inactive gonad seem to point to a similar mechanism controlling the germline. For example, *ccr4-not transcription complex subunit 10*, is a gene known to be part of the deadenylase complex targeting mRNAs recruited by PUF proteins, resulting in shorter poly(A)-tails that are unstable or translationally repressed, and *elongation factor 1 alpha* is known to form a translation repression complex together with PUF and Ago proteins [115]. Another interesting transcript found in the inactive gonad was *btg member 2*, a Tob/BTG protein that in vertebrates works in growth control and differentiation, and in *C. elegans* encodes for the germline specific FOG-3 (whose name derives from the phenotype of mutants: **f**eminization **o**f **g**ermline) that regulates germ cells to adopt the sperm fate [116]. Additionally, a *transcription factor btf3 homolog 4* (*bicaudal-C* in *Drosophila*), that encodes an RNA-binding protein implicated in germline control, which regulates expression of germline-specific mRNAs poly(A)-tail length by recruiting CCR4-NOT deadenylase [117], was also found in the inactive gonad.

Two other transcription factors were also found involved in differentiation pathways: *transcription factor hes-1* and *transcription factor 21*. *Tcf21* is a basic helix-loop-helix gene that in rats has been identified as a direct downstream target of SRY, the testis determining gene that promotes Sertoli cell differentiation in rats [118]. The *hes-1* (*hairy and enhancer of split 1* gene in *Drosophila*), is involved in negative regulation of transcription, and has been found to have a stage-dependent expression also in Sertoli cells [119]. One last transcript found in the inactive gonad library was the *nuclear receptor subfamily 0 group b member 1*, also known as *dax-1* (*dosage-sensitive sex reversal, adrenal hypoplasia critical region on the X chromosome, gene 1*), after the mutant phenotype that causes an X-linked form of adrenal hypoplasia in human males. This gene is known to be a negative regulator of steroid hormone production by transcriptional repression of genes involved in the steroidogenic pathway [120], and to also antagonize and inhibit the function of the androgen receptor (AR), possibly to modulate transcription of AR-dependent genes [121]. Taken together, these results evidence that the maintenance of germline through the transcription of the discussed genes, is an essential process even during the resting period of the gonad after spawning.

### SSR in coding sequences

Simple-sequence repeats (SSRs) have increasingly become the marker of choice for population genetic analyses. EST-SSRs have the advantage of being higher conservation across species than anonymous genomic SSRs, and hence are more transferable across taxonomic boundaries for genome mapping and comparative structural genomics [122], [123]. Indeed, two of the EST-SSR identified in this work produced hits with two microsatellites described for other pectinid species, *Argopecten purpuratus* microsatellite B119 (Genbank accession JN799265.1) and *Chlamys farreri* microsatellite clone CFFD148 (Genbank accession EF148946.1) (Table S5). Besides mapping, polymorphic SSRs in coding genes are of interest in a physiological context and hence considered as non-neutral for selection, since altered promoter expression and altered 3' stability, and slippage of transcription, might be affected by changes in repeat numbers [122], [124]. Some of the microsatellites for which an annotation of the nucleotide sequence was obtained were found to be located in the 3' UTR of mRNAs, as a tri-repeat of TAT with a flanking region similar to the troponin I of *Patinopecten yessoensis* cDNA (Genbank accession AB008006.1). Expansions or contractions of SSRs located in the coding region can lead to frame-shift mutations, premature stop codons and changes of amino acid sequences, although the most common repeat in the coding region of mRNAs is the trinucleotide (or their multiples). The region with the highest densities of trinucleotide repeats is the 5' UTR [125]. The microsatellites identified in this study increase the panel of polymorphic microsatellites previously reported by [126] and [127] for the species, and could help to refine the genetic map in which 22 linkage groups were obtained in the consensus map [72], while the haploid chromosome number is 19 [128]. The newly identified microsatellites included only 10 that were identified as polymorphic; the reason could be that the 23 individuals used to construct the libraries were derived from one family of half-sibs produced by fertilizing oocytes from one scallop with mixed sperm of three other scallops.

In conclusion, the discovery of genes with inferred function in meiosis, recombination, and DNA repair is the first step towards the understanding of triploid-induced sterility. It also allows the identification of transcriptional markers for meiosis and spermatogenesis, and provides a panel of genes to perform functional characterizations by reverse genetics techniques like RNA interference, a tool already used in marine bivalves during the gametogenic process [129].

## Supporting Information

Table S1 Primer sequences for qPCR analyses of gene expression on selected genes.
(PDF)

Table S2 Top blastx hits for library-specific transcripts (sheet 1 and 2), and for transcripts represented by reads obtained from both libraries (sheet 3).
(XLS)

Table S3 Ontology terms (GO terms) associated to library-specific transcripts (sheet 1 and 2), and for transcripts represented by reads obtained from both libraries (sheet 3).
(XLS)

Table S4 Enrichment analysis of ontology terms between the two libraries, by Fisher-exact test using the Blast2GO software.
(XLS)

Table S5 Microsatellites found *in silico* on expressed sequences with potential primer sequences. PCR amplification.
(XLS)
