# Supplementary material for: Identification of Male Gametogenesis Expressed Genes from the Scallop Nodipecten subnodosus by Suppressive Subtraction Hybridization and Pyrosequencing
Source: PLoS One. 2013 Sep 16;8(9):e73176. doi: 10.1371/journal.pone.0073176 (PMC3774672; doi:10.1371/journal.pone.0073176)
Supplement: Table S1 — Primer sequences for qPCR analyses of gene expression on selected genes. (PDF) [file pone.0073176.s001.pdf]

Supplementary file 1. RACE and qPCR primers used in this study.

Gene	Direction	Sequence
meiotic recombination protein DMC1/LIM15 homolog (*dmc1*)		
	Fwd	5'-AGGTGCCAATGGTTATACCG-3'
	Rev	5'-TTGAAGACACCTGGCTCCTC-3'
DNA repair protein RAD51 homolog 3 (*rad51*)		
	Fwd	5'-CTTCACCCATTCGCACTTTT-3'
	Rev	5'-TAGCGTGGCCTTTCACTTTC-3'
synaptonemal complex protein 3 (*scp3*)		
	Fwd	5'-CGCAGTTTTCAACCAGTGGGAAG-3'
	Rev	5'-TTGTGGCAGGTCCCAAGATCC-3'
HORMA domain containing protein 1 (*horma*)		
	Fwd	5'-TTTCAAGCCAGCACAGAGTG-3'
	Rev	5'-TTCCTGCACAGGACTCTCAA-3'
e3 ubiquitin protein ligase CCNB1IP1 (*e3-ccnb1ip1*)		
	Fwd	5'-TTTGCTTGGGTGACATCTTG-3'
	Rev	5'-ACTGCTCATTTGGCTGAAGA-3'
p33 RINGO		
	Fwd	5'-ATGGGCTCTTGGTCACAAATGG-3'
	Rev	5'-CCATTATCTCTTCACAGCATCGCT-3'
